# Heart-healthy diets including phytostanol ester consumption to reduce the risk of atherosclerotic cardiovascular diseases. A clinical review

**DOI:** 10.1186/s12944-024-02330-7

**Published:** 2024-10-21

**Authors:** Piia Simonen, Lotta Nylund, Erkki Vartiainen, Petri T. Kovanen, Timo E. Strandberg, Katariina Öörni, Ingmar Wester, Helena Gylling

**Affiliations:** 1 Heart and Lung Center, Cardiology, Helsinki University Hospital, University of Helsinki, Helsinki, Finland; 2 Raisio Group plc, Raisio, Finland; 3 Department of Public Health and Welfare, Finnish Institute for Health and Welfare, Helsinki, Finland; 4 Wihuri Research Institute, Helsinki, Finland; 5 Helsinki University Hospital and University of Helsinki, Helsinki, Finland; 6 Center for Life-Course Health Research, University of Oulu, Oulu, Finland

**Keywords:** Atherosclerosis, Cholesterol, Cholesterol absorption, Coronary artery disease, LDL-cholesterol, LDL aggregation, Phytostanol- and phytosterol ester

## Abstract

The risk of atherosclerotic cardiovascular diseases (ASCVDs) can be reduced by lowering low-density lipoprotein cholesterol (LDL-C) concentrations. Nevertheless, ASCVDs still cause most deaths worldwide. Here, we discuss the prevention of ASCVD and the event risk with a focus on heart-healthy diets, i.e., low intakes of saturated and trans-fatty acids and cholesterol, and high intakes of unsaturated fatty acids, viscous fibre, and dietary phytostanols as fatty acid esters, according to international dyslipidaemia treatment guidelines. Calculations based on both FINRISK and Cholesterol Treatment Trialists’ Collaborators regression equations indicate that heart-healthy diets combined with phytostanol ester reduce LDL-C concentrations to such an extent that the 10-year estimated reduction in the incidence of coronary artery disease would be 23%. This information can be used, in particular, to prevent the development of subclinical atherosclerosis in healthy middle-aged populations and the progression of atherosclerosis to ASCVD. The outcome of simple and feasible dietary changes, and, when needed, combined with statins, can be significant: reduced mortality, an increased number of healthy life-years, and reduced healthcare costs.

## Introduction

Atherosclerotic cardiovascular diseases (ASCVDs), especially coronary artery disease (CAD), are the most common causes of death worldwide [1], and the prevalence of CAD is expected to increase with aging of the populations. In men and women aged 15–49 years, cardiovascular disease (CVD) mortality increased in 1990–2019 by 25% worldwide, and CAD and stroke were the leading causes of death even in these age groups [2, 3]. In addition to ASCVDs, early-onset asymptomatic atherosclerosis, also called subclinical atherosclerosis, is common in apparently healthy middle-aged people, and it may progress to ASCVD in a relatively short time, on its own part accounting for the high prevalence figures [4, 5], calling for dietary and life-style changes earlier in life to lower circulating low-density lipoprotein (LDL) cholesterol (LDL-C) concentrations.

Of the apoprotein B100 (apoB)-containing lipoproteins, mainly LDLs cause ASCVDs [6, 7]. In fact, the effect of the elevated concentrations of LDL-apoB and LDL-C is cumulative- the longer the time of exposure, the greater the ASCVD risk [8]. Decreasing circulating LDL-C concentrations has been shown to decrease the risk of ASCVD events in epidemiologic, genetic, Mendelian randomization, and randomized clinical studies [6, 7, 9, 10]. In the clinical studies, LDL-C concentrations were reduced by upregulating the expression of hepatic LDL-apoB receptors by means of dietary changes (low-fat and low saturated fat diets), ileal bypass operations, or by statin, resin, or ezetimibe treatment, leading to reduction of ASCVD events [6, 7, 9, 10]. The results of a study by Silverman et al. [10] indicated that the reduced risk of ASCVD events is not dependent on the pleiotropic properties of statins. Thus, there are simple, feasible, and effective dietary and lifestyle means available to reduce the risk of ASCVD events, but plausibly they are markedly underutilized, since the prevalence and the event risks of ASCVDs have not been diminished.

In the European dyslipidaemia treatment guidelines, changes in dietary habits towards heart-healthy diets are recommended as the primary steps of LDL-C lowering in the general population, in primary prevention, and in individuals at low risk of ASCVDs, while in hypercholesterolaemic individuals, dietary changes are recommended in combination with lipid-lowering medication, depending on the ASCVD risk level [8]. Heart-healthy diets include low intakes of saturated fatty acids (SFAs), trans-fatty acids, and cholesterol, and high intakes of unsaturated fatty acids and viscous fibre, potentially combined with the intake of phytostanols/phytosterols as fatty acid esters.

Other risk factors may also influence the development of atherosclerosis. First, in addition to the LDL-apoB lipoproteins, also the other apoB-containing lipoproteins, which increase serum triglyceride concentrations, and the elevated serum lipoprotein(a) (Lp(a)) levels are considered atherogenic [11–13]. Serum triglyceride concentrations can be reduced by dietary means e.g., with phytostanol or phytosterol ester supplementation [11]. In a meta-analysis with 17 studies and 23 study arms consumption of $\:\ge\:$ 2 g phytostanols or phytosterols/day over eight weeks significantly lowered not only serum total and LDL-C concentrations but also those of serum triglycerides by -3.77 mg/dL, 95% confidence interval (CI), -6.04, -1.51, *P* = 0.001 [11]. Regarding elevated serum Lp(a) concentration, its reduction with dietary means is problematic. Lowering the intake of SFAs increases the concentration of Lp(a), whereas a diet low in carbohydrates and high in SFAs decreases its concentration [13].

Second, aggregation susceptibility of LDL particles [14] and high cholesterol absorption efficiency increase the risk of atherosclerosis [15–18]. To this end, the roles of dietary SFAs, cholesterol, viscous fibre, and phytostanol esters on LDL-C concentrations and the estimated risk of ASCVD are first briefly discussed, and three types of heart-healthy dietary patterns, i.e., multifunctional diets, the Mediterranean diet, and a comparison of Mediterranean and phytostanol ester diets are introduced to clarify their efficacy in modifying circulating risk factors and reducing the risk of ASCVD events. Second, new calculations for ASCVD risk reduction by changes in dietary fat combined with the use of phytostanol esters are presented. Third, the possibility of reducing the atherogenic potentials of LDL particles and cholesterol metabolism by dietary means in high cholesterol absorbers is discussed.

## Heart-healthy diets

### Dietary fat, cholesterol, and viscous fibre

The role of SFAs in the development of ASCVDs has been effectively investigated, but the consensus is not without controversies [19]. However, in international dyslipidaemia treatment guidelines, the consensus is that by replacing dietary SFAs with monounsaturated fatty acids (MUFAs) and n-6 polyunsaturated fatty acids (n-6 PUFAs), LDL-C concentrations can be reduced by approximately 5–10% from baseline or versus controls [8, 19, 20]. In addition, fatty acids can remodel the plasma lipidome and in that way also interfere with the development of atherosclerosis. Thus, replacement of dietary SFAs with MUFAs and n-6 PUFAs essentially changed the fatty acid composition in the plasma lipidome [21]. High intakes of MUFAs and n-6 PUFAs particularly reduced the levels of glycerolipids and sphingolipids, which were related to higher risks of ASCVDs, and increased the levels of lipids predicting lower risk.

A high-cholesterol diet increases serum cholesterol and LDL-C concentrations. Individual responses, however, vary, and a cholesterol challenge does not invariably result in the elevation of serum and lipoprotein cholesterol concentrations [22]. In a population-based meta-regression analysis, LDL-C concentrations increased by about 0.12 mmol/L (4.6 mg/dL) for each additional 100-mg increase in dietary cholesterol/day [23, 24].

Oat and barley contain β-glucan, a viscous fibre, which reduces the absorption of cholesterol and especially the reabsorption of bile acids. As a consequence, the concentrations of serum cholesterol and LDL-C are reduced. For example, 3–4 g/day of β-glucan from 80 g of oat and barley flakes reduced LDL-C concentrations by approximately 0.21–0.33 mmol/L from baseline values (*P* < 0.05) [25].

### Dietary phytostanol and phytosterol esters

Phytostanols and phytosterols are naturally present in plant-based foods, especially in vegetable oils, spreads and margarines, breads, cereals, vegetables and fruits [26]. The mean daily intake of naturally occurring phytosterols in a Western diet is about 300 mg, but the amount of phytostanols is much lower, less than 24 mg per day [26]. In practice the naturally occurring phytostanols and phytosterols have no effect on serum total and LDL-C concentrations. In a controlled study in healthy subjects, low intake (126 mg phytosterols/2000 kilocalories/day) or high intake (449 mg phytosterols/2000 kilocalories/day) did not affect plasma LDL-C concentrations [27]. The main difference between phytostanols and phytosterols is that the absorption efficiency of phytostanols in humans is very low, less than 0.2%, and consequently their circulating levels are also low, less than 0.3 µmol/L, approximately 10 000-fold lower than that of LDL-C concentration [26]. The absorption efficiency of phytosterols is less than 2%, and their circulating levels are less than 24 µmol/L, respectively.

Phytostanols as fatty acid esters were developed in 1989 and phytosterol esters in the early 2000s to reduce the absorption of cholesterol and thus provide a dietary means to enhance LDL-C reduction. Their addition to the diet should be particularly considered if LDL-C goals are not reached in individuals with a low ASCVD risk, or as an adjunct to pharmacological therapy in high- and very-high-risk patients who fail to achieve LDL-C goals on statins [8]. In the following we will focus on phytostanol esters as part of heart-healthy diets because of the ample availability of dietary experiments and because most of the information obtained can be utilized also for phytosterols.

**Fig. 1:**
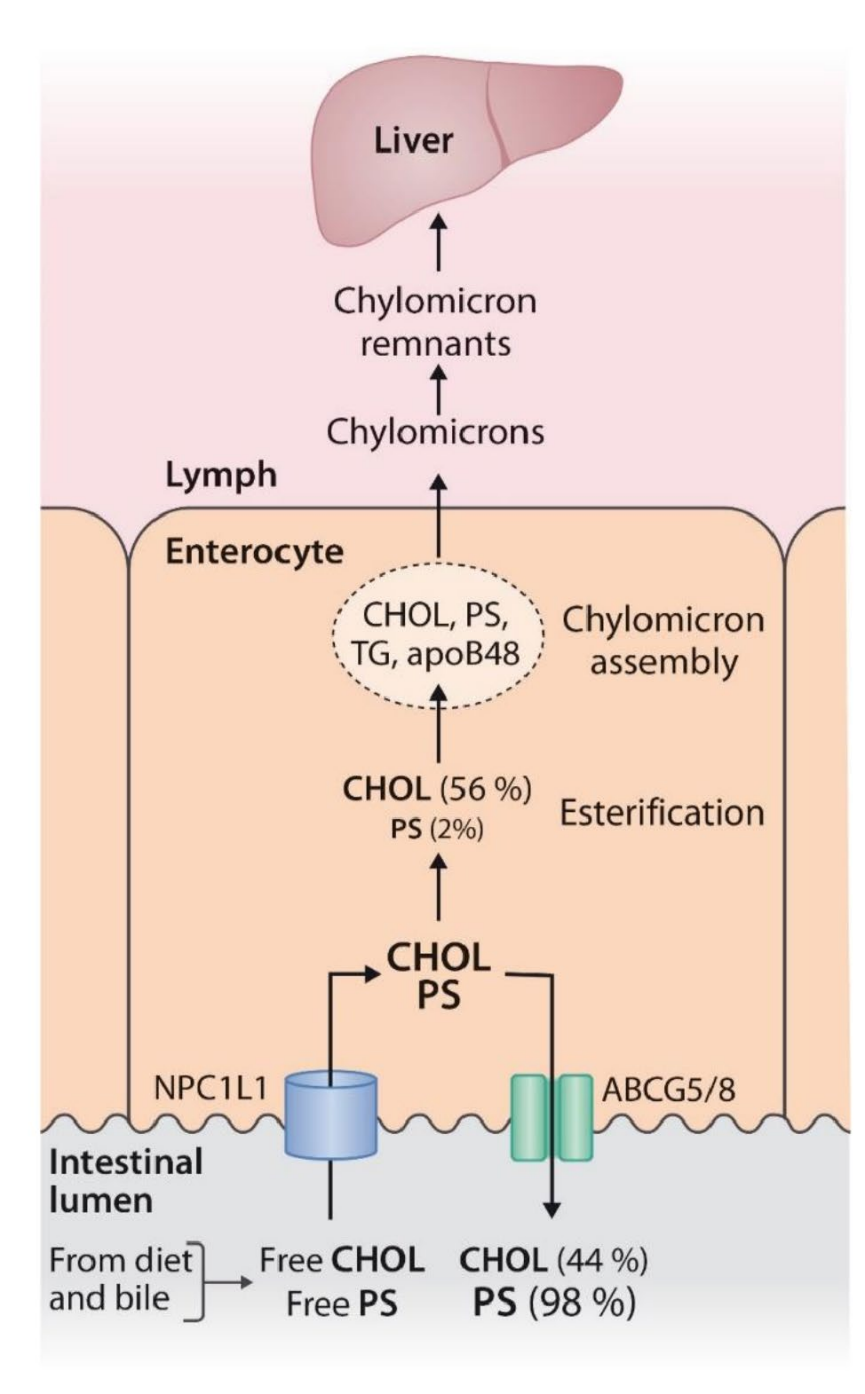
Simplified scheme of the absorption of cholesterol, phytosterols, and phytostanols Footnote: Niemann–Pick C1-Like 1 transporter takes up cholesterol, phytosterols, and phytostanols into the enterocyte, but about half of the cholesterol and most of the phytosterols, and phytostanols in particular, are driven back to the intestinal lumen via the adenosine triphosphate-binding cassette (ABC) transporters G5 and G8. Eventually, approximately 50–60% of the cholesterol, 0.5–2% of the phytosterols, and 0.04–0.15% of the phytostanols are absorbed into the body [26] *Abbreviations*: ABCG5/8 = adenosine triphosphate-binding cassette (ABC) transporters G5 and G8, apoB48 = apoprotein B48, CHOL = cholesterol, NPC1L1 = Niemann–Pick C1-Like 1 transporter, PS = phytosterols and phytostanols, TG = triglycerides

In the proximal small intestine, pancreatic cholesterol esterase cleaves the ester bond between fatty acids and phytostanol, after which free phytostanol displaces cholesterol from the mixed micelles [28]. The displaced cholesterol is not absorbed but excreted into the faeces (Fig. 1).

**Fig. 2:**
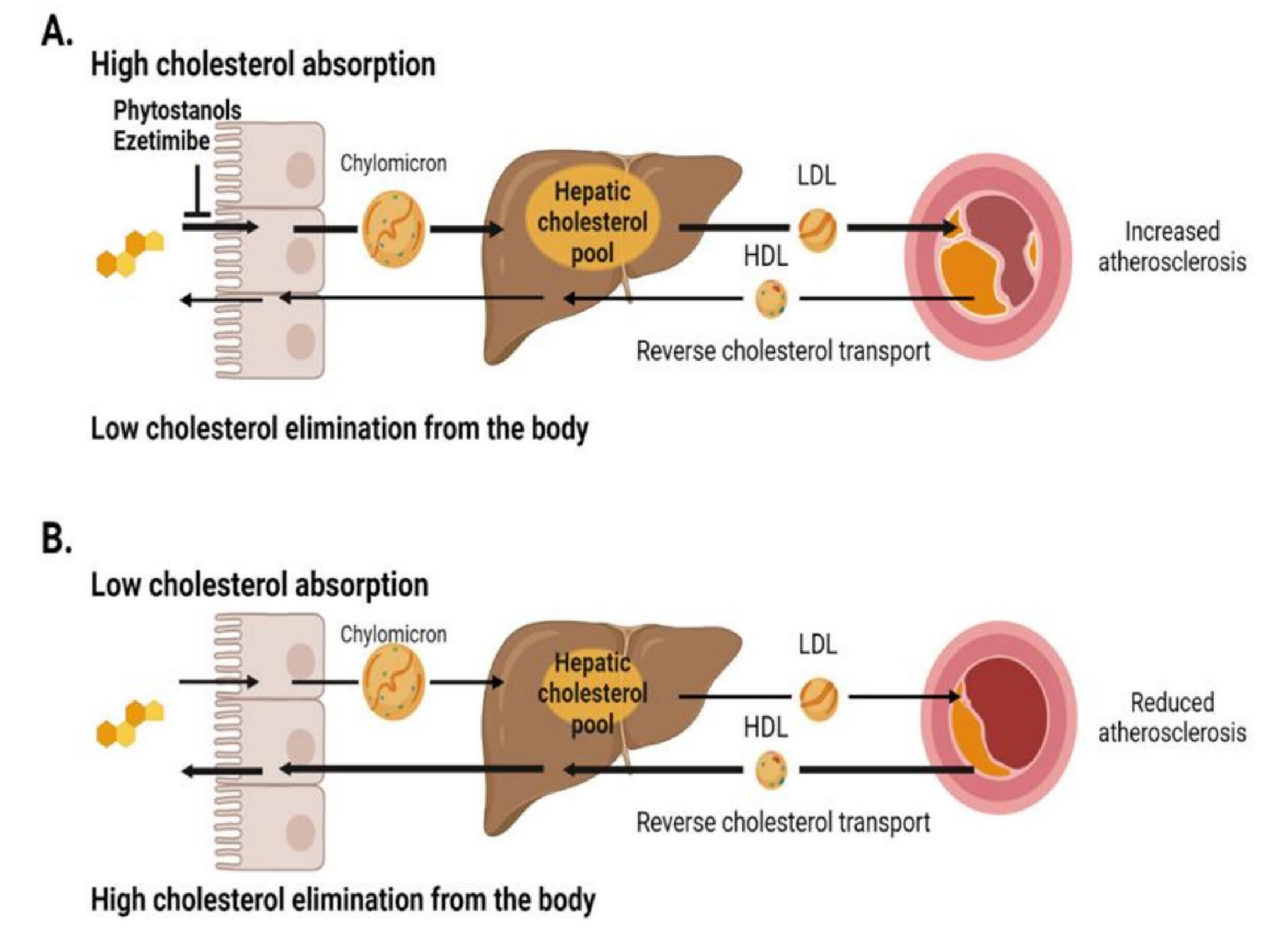
Cholesterol metabolism including cholesterol absorption from the small intestine and transport into the liver, delivery into tissues, and elimination from the body mainly via bile in individuals with high (**panel A**) and low (**panel B**) cholesterol absorption efficiency Footnote: The risk of atherosclerosis is increased in high vs. low cholesterol absorption, which can be interfered with by reducing cholesterol absorption by dietary and pharmacological means, resulting in increased cholesterol elimination from the body *Abbreviations*: LDL = low-density lipoprotein, HDL = high-density lipoprotein

Phytostanols reduce the absorption of cholesterol by approximately 50–60% [26, 29]. Less cholesterol is transported to the liver, the hepatic cholesterol pool is diminished, and the expression of hepatic LDL-apoB receptors is upregulated. Cholesterol efflux from tissues is activated, cholesterol elimination from the body to the faeces via bile is increased, LDL-C concentrations are reduced, and cholesterol metabolism is modified to become less atherogenic [15–18] (Fig. 2). The LDL-C reductions vary between individuals reflecting the differences in lipoprotein and cholesterol metabolism.

Based on the results of a meta-analysis, a daily intake of 2–3 g of phytostanols as fatty acid esters decreased LDL-C levels on average by 0.33–0.42 mmol/L (9–12%) [30]. Phytostanol esters also lower the concentrations of serum phytosterols and non-high-density lipoprotein cholesterol (non-HDL-C) but they do not in general affect the concentrations of high-density lipoprotein cholesterol (HDL-C), serum triglycerides, Lp(a), or proprotein convertase subtilisin/kexin type 9 (PCSK9) [29, 31, 32]. Even though different conditions such as overweight and obesity interfere with cholesterol metabolism, the cholesterol-lowering efficacy of phytostanol esters is not affected in these conditions [33]. In addition, phytostanol esters similarly decrease serum and lipoprotein lipids in persons with low and high cholesterol absorption efficiency [29]. For example, in one study, LDL-C concentrations decreased by 0.37 ± 0.09 mmol/L (mean ± standard error (SE)) in low cholesterol absorbers and by 0.52 ± 0.11 mmol/L in high absorbers (not significant (NS) between the groups) [29].

## Heart-healthy dietary patterns

### Multifunctional diet

Extensive dietary changes are often needed to reduce LDL-C concentrations meaningfully. We first describe the structure and efficacy of a so-called multifunctional diet on serum and lipoprotein lipids [34]. In this randomised, controlled, eight-week intervention the dietary changes included food items with low contents of SFAs and cholesterol, low glycaemic index, and high contents of viscous fibre, PUFAs, and phytostanol esters (2–2.7 g phytostanols/day as fatty acid esters). The control group continued their habitual home diets without phytostanol esters. The participants were otherwise healthy, but they were slightly hypercholesterolaemic without lipid-lowering medication.

In the study, LDL-C concentrations significantly decreased from their baseline values by 35% (~ 1.4 mmol/L), and the decreases in plasma cholesterol and LDL-C concentrations also differed significantly from those in the control group (*P* < 0.001) [34]. HDL-C and plasma triglyceride concentrations decreased from baseline by 11% and 16% (*P* < 0.05 for both). In the control group, only HDL-C concentrations decreased from baseline, by 5% (*P* < 0.05).

The marked reduction of LDL-C concentrations in the multifunctional diet group would be expected to reduce the risk of ASCVD events, since both a low fat/low saturated fat diet [10] as well as phytostanol ester consumption [35] lower LDL-C concentrations by upregulating the expression of hepatic LDL-apoB receptors, as found in large clinical trials [9, 10]. A calculated estimate of the 10-year risk score for CAD indicated a 36% risk reduction (*P* < 0.0001) in the multifunctional diet group, whereas in the control group the risk estimate remained unchanged [34].

### Mediterranean diets

In a randomized, controlled sub-study of the Preventión con Dieta Mediterránea (PREDIMED) study, over 700 asymptomatic persons at a high risk of CVD participated in a three-month intervention trial, consuming two types of Mediterranean diet or a low-fat control diet [36]. The Mediterranean diets consisted mainly of fresh fruit, vegetables, fatty fish and seafood, legumes, white meat, wine with meals, and either extra-virgin olive oil, or nuts and peanuts. The low-fat control diet consisted of low-fat dairy products, bread, potatoes, pasta, rice, fresh fruit, vegetables, and lean fish and seafood. The Mediterranean diets significantly decreased LDL-C levels by 0.10–0.15 mmol/L from baseline, and there was a similar reduction in the control group. However, the Mediterranean diets were also significantly associated with decreased blood pressure, and decreased levels of blood glucose, serum insulin, and C-reactive protein, and increased HDL-C concentrations. The reduction of blood glucose and serum insulin levels may reflect not only the dietary changes but also the type of the study populations; in both the main PREDIMED study and the sub-study approximately half of the participants had type 2 diabetes, and their mean body mass indices were from 29.4 to 30.2 kg/m^2^. 90% of the study populations were overweight or obese [36, 37].

In the main PREDIMED study the effect of a Mediterranean diet on the risk of CVD events was investigated in 7447 asymptomatic persons, and the median follow-up period was 4.8 years [37]. The participants were similarly randomized into three groups as in the PREDIMED sub-study [36]. The CVD event risk was significantly lower in both Mediterranean diet groups versus the control group. In the combined Mediterranean diet groups the 5-year absolute risk of a CVD event was 3.6% CI (3.0–4.3) vs. 5.9% CI (4.8–7.2) in the control group.

### Mediterranean diet compared with phytostanol ester consumption

In a third intervention trial lasting for four months the effects of a Mediterranean diet on vascular risk factors and CVD event risk were compared between three groups after a run-in period on a Step-1 hypolipidemic diet: a Mediterranean diet group (*n* = 50), a phytostanol ester group (*n* = 50), and a control group using placebo spread (*n* = 50) [38]. In the Mediterranean diet group the aim was to improve adherence to the Mediterranean diet [39], whereas in the phytostanol ester and control groups the participants followed the Step-1 hypolipidemic diet. The phytostanol dose was 2 g/day of phytostanols as fatty acid esters. All participants were mildly hypercholesterolaemic without ASCVD or diabetes. The mean body mass index was similar between the groups, from 27.3 to 27.9 kg/m^2^ (NS).

In the control group, none of the cardiovascular risk factors changed during the intervention period [38]. In the Mediterranean diet group, the concentrations of LDL-C and serum triglycerides were significantly decreased from baseline by 9% and 6%, and HDL-C concentrations were increased by 6%. Plasma glucose, fibrinogen, and plasminogen activator inhibitor type 1 activity were also significantly reduced.

In the phytostanol ester group, after four weeks, LDL-C concentrations were already significantly reduced by 16% from baseline and this reduction was significantly greater than those in the control (-2%) and Mediterranean diet (-9%) groups [38]. Circulating levels of high sensitivity C-reactive protein decreased similarly and significantly from baseline in the Mediterranean diet (-19%) and phytostanol ester (-17%) groups.

Regarding the estimated CVD risk, there was no change in the control group, but in the Mediterranean diet and phytostanol ester groups the estimated CVD risks were reduced similarly, depending on the risk assessment methodology, from 24 to 32% in the Mediterranean diet group and from 26 to 30% in the phytostanol ester group [38].

Accordingly, by way of dietary means it is possible to interfere with the development of atherosclerosis and reduce the risk of ASCVD events. With the different kinds of diet it seems possible to influence various circulating risk factors.

### New calculations for ASCVD risk reduction by way of heart-healthy diets including phytostanol esters

According to the Keys, Anderson and Grande formula [40], reducing the intake of SFAs in Finns from the current 15 to 10 energy% and increasing the intake of PUFAs from 7 to 10 energy% would reduce serum total cholesterol concentrations by 0.40 mmol/L. Interestingly, the use of phytostanol ester margarine reduced serum total cholesterol concentrations by 0.60 mmol/L (10%) in a one-year intervention [41].

Based on Finnish FINRISK 10-year risk calculations [42], a change in the fat quality of a standard diet could result in an 11% decrease in the 10-year incidence of CAD. Strikingly similar results were observed in a Norwegian study, where changes in dietary fat quality lowered serum cholesterol concentrations by 0.44 mmol/L and decreased the estimated risk of ASCVD by 8% [43, 44]. Regarding the use of phytostanol ester products, FINRISK calculations revealed that they could reduce the incidence of CAD by 15%. When both changes in dietary fat and the use of phytostanol ester products are implemented, the reduction in the incidence of CAD could be 23%. The LDL-C lowering effect through the use of phytostanol esters is additive to that of recommended diets [45].

Both the phytostanol ester supplementation and ezetimibe treatment interfere with cholesterol absorption. A randomized controlled study with ezetimibe 10 mg/day alone or combined with 2.5 g/day of phytosterol supplementation demonstrated that the combination therapy had an additive effect on LDL-C lowering and it also modified cholesterol metabolism [46]. Thus, ezetimibe alone lowered the LDL-C concentration from the baseline control value of 129 mg/dL to 108 mg/dL (*P* < 0.01), which still was lowered by the ezetimibe-phytosterol combination therapy to 101 mg/dL (*P* < 0.05). In addition, the ezetimibe-phytosterol combination significantly lowered cholesterol absorption and increased cholesterol excretion from the body via bile compared with the baseline control- and ezetimibe alone values (*P* < 0.001) reducing the atherosclerosis burden in the body [15–17].

In addition, based on the results of a meta-analysis of phytostanol ester studies [30], a daily dose of 3 g of phytostanols as esters reduced LDL-C concentrations by 0.42 mmol/L (12%). When placed in the ASCVD risk equation of the Cholesterol Treatment Trialists´ Collaborators [9], this resulted in a reduction in the 5-year risk of ASCVD events by approximately 9% [31]. Thus, the above calculations reveal that dietary changes including phytostanol esters as part of a heart-healthy diet (in combination with statins when needed) offer an effective means of reducing the risk of ASCVD events both at the population level and in subjects with different risk levels of ASCVD.

### Heart-healthy diets and LDL aggregation susceptibility

Increased aggregation susceptibility of LDL particles has been found to increase the risk of ASCVD events independently in patients with CAD and peripheral artery disease [14, 47]. In order to clarify the potential mechanisms behind aggregation susceptibility, the effects of dietary MUFAs, PUFAs, SFAs, carbohydrates, and phytostanol esters on LDL aggregation and LDL binding to proteoglycans (another atherogenic variable) were evaluated in four randomized, controlled clinical trials [48–51]. High intakes of MUFAs and PUFAs decreased LDL aggregation susceptibility and LDL binding to proteoglycans, whereas a high intake of SFAs increased LDL aggregation [48–50]. Intake of carbohydrates had no effect on LDL aggregation. LDL aggregation susceptibility correlated with the alterations in LDL lipids; it correlated positively with the proportions of total sphingomyelin and negatively with the proportions of several phosphatidylcholines and triglycerides in the LDL particles.

In addition, phytostanol ester consumption (3 g/day of phytostanols as fatty acid esters) was found to reduce LDL aggregation susceptibility and the binding of plasma lipoproteins to proteoglycans [51]. The changes in LDL aggregation correlated with the alterations of LDL surface lipids, so that decreased LDL aggregation susceptibility was associated with a decreased proportion of LDL-sphingomyelins and an increased proportion of LDL-triglycerides. The decrease in LDL aggregation was stronger in normal-weight subjects than in overweight and obese individuals. In addition, increased LDL aggregation and a more aggregation-prone LDL lipidome were present in individuals with high vs. low baseline cholesterol absorption efficiency [52].

### Heart-healthy diets and high cholesterol absorption efficiency

The absorption of cholesterol is mainly genetically regulated by the intestinal transporters Niemann–Pick C1-like 1 (NPC1L1) transporter and the adenosine triphosphate-binding cassette (ABC) transporters ABCG5/G8 [18] (Fig. 1). The loss-of-function (LoF) variations of these transporters have demonstrated the connections between cholesterol metabolism and ASCVDs [18]. LoF variations in NPC1L1 reduce cholesterol absorption, whereas LoF variations in (ABC) G5 and G8 increase cholesterol absorption. Cholesterol absorption is considered high when its absolute efficiency is > 50–60%, and it occurs approximately in one third of populations [53, 54].

Is it possible to modify cholesterol metabolism by dietary means so as to be less atherogenic in high cholesterol absorbers? The only dietary evidence is obtained from phytostanol ester consumption of 2–3 g of phytostanols/day (Fig. 2). This reduced cholesterol absorption efficiency by 41% and 47% in low- and high cholesterol absorbers (*P* < 0.001 for both, NS between groups), resulting in LDL-C reductions of 0.37 mmol/L and 0.52 mmol/L in low vs. high cholesterol absorbers (NS between groups), respectively [29]. As a consequence, cholesterol elimination from the body to the faeces via bile as faecal neutral sterols increased by 27% in the low absorbers vs. 47% in the high absorbers (*P* < 0.001 between groups) [29], decreasing the atherosclerotic burden by enhanced elimination of cholesterol from the body [15–17]. Thus, it is obvious that phytostanol ester consumption alone, and plausibly even more effectively in combination with heart-healthy diets or combined with the ezetimibe treatment [46], modifies cholesterol metabolism so as to become less atherogenic, especially in high cholesterol absorbers.

### Study strengths and limitations

According to the lipid lowering guidelines, dietary modifications are the cornerstone to start controlling the circulating concentrations of LDL-C and modifying the cholesterol metabolism less atherogenic to prevent the development and reduce the event risks of ASCVDs. The strengths of this study are that an extensive amount of detailed high-quality information is available of dietary means alone or combined with lipid-lowering drugs to prevent ASCVDs.

The limitation of the study is that the impact of e.g., dietary fatty acids on LDL-C concentrations and ASCVD risk has provoked debates of controversies and conflicting results. In this study with a focus on clinical interventions the attention was paid to the consensus of the dietary plans in the international dyslipidaemia treatment guidelines. Thus, the controversies were not discussed in this context.

## Conclusions

A few but most essential dietary factors and three heart-healthy diets on LDL-C concentrations and on the risk of ASCVD events was discussed. LDL-C was reduced by 35%, approximately 1.4 mmol/L with clinical relevance. LDL-C reduction 1 mmol/L leads to a reduction of ASCVD events by 21% [10]. The preventive effects are additional combining diet and lipid lowering drugs when needed. The diets also modify the metabolism of cholesterol, serum lipidome, and the aggregation of LDL particles less atherogenic. The future perspective is to expand the understanding of the atherogenic traits in the lipid metabolism and their prevention.

## Data Availability

No datasets were generated or analysed during the current study.

## Abbreviations

ABCG5/G8: Adenosine triphosphate-binding cassette (ABC) transporters G5 and G8
ApoB: Apoprotein B
ASCVD: Atherosclerotic cardiovascular disease
CAD: Coronary artery disease
CI: Confidence interval
CVD: Cardiovascular disease
HDL-C: High-density lipoprotein cholesterol
LDL: Low-density lipoprotein
LDL-apoB: Low-density lipoprotein apoprotein B100
LDL-C: Low-density lipoprotein cholesterol
LoF: Loss-of function
Lp(a): Lipoprotein(a)
MUFAs: Monounsaturated fatty acids
non-HDL-C: Non-high-density lipoprotein cholesterol
NPC1L1: Niemann–Pick C1-like 1 transporter
NS: Not significant
PCSK9: Proprotein convertase subtilisin/kexin type 9
PREDIMED: Preventión con Dieta Mediterránea
PUFAs: n-6 polyunsaturated fatty acids
SE: Standard error of mean
SFAs: Saturated fatty acids
